# Speckle reduction in double-pass retinal images

**DOI:** 10.1038/s41598-019-41168-3

**Published:** 2019-03-14

**Authors:** Donatus Halpaap, Carlos E. García-Guerra, Meritxell Vilaseca, Cristina Masoller

**Affiliations:** 1 Departament de Física, Universitat Politècnica de Catalunya, St. Nebridi 22, 08222 Terrassa Barcelona, Spain; 2 Centre de Desenvolupament de Sensors, Instrumentació i Sistemes, Universitat Politècnica de Catalunya, St. Nebridi 10, 08222 Terrassa Barcelona, Spain

## Abstract

The double pass (DP) technique quantifies the optical quality of the eye by measuring its point spread function. The low reflectivity of the retina requires the use of a high-brightness, point-like illumination source, and thus, DP systems use laser diodes (LDs). However, LDs light produces speckle, and a low-cost solution to reduce speckle is to include a vibrating mirror in the beam path. With the goal of finding an all-optical solution, here we perform a comparative study of the amount of speckle produced by three semiconductor light sources: an LD, a light emitting diode (LED), and a superluminescent diode (SLED). We also compare the results with the speckle reduction that is obtained with a vibrating mirror. We find that the SLED is a good alternative to LD illumination, as the amount of speckle in the image is almost as low as that obtained with an LD and a vibrating mirror in the beam path.

## Introduction

The double pass (DP) technique offers an objective evaluation of the overall optical quality of the eye (ocular aberrations and intraocular scattering)^1–7^. It is based on imaging a point source onto the retina, where the light is reflected and recorded after its second pass through the ocular media. From the DP image the ocular point spread function (PSF, that is the impulse response of the optics, i.e., the image of a point source) and the modulation transfer function (MTF, that is the Fourier transform of PSF) can be obtained, which are important measures of the quality of an optical system. DP imaging is routinely used for comparative studies, e.g., after refractive surgery^8^ or for cataract classification^9,10^.

In order to accurately determine the PSF, a DP system requires a point-like light source. Due to the low reflectivity of the retina, the light source used should also emit high enough power. Therefore, laser diodes (LDs) are commonly used, with the wavelength and the emitted power limited by the patient’s safety and comfort. However, coherent monochromatic laser light, and the relative roughness of the human retina on the scale of the wavelength, lead to speckle formation, which degrades the DP image, altering the determination of the parameters that characterize the optical properties of the eye.

Speckle is an optical artifact of coherent waves interfering with each other that is often undesired in imaging. Speckle can be reduced by using an illumination that is spatially or temporally partly incoherent^11^. Spatial coherence refers to a fixed phase relationship between different points of a wavefront. It can be reduced by introducing a moving diffuser into the beam in order to modulate it randomly^11^. Temporal coherence is related to the spectral width of a source and broadband illumination reduces the problem of speckle^12^.

However, in DP systems, mainly due to longitudinal chromatic aberrations of the eye, white-light illumination sources lead to an underestimation of the MTF^13^. Therefore, alternative speckle-reduction solutions have been proposed in the literature, like acoustic modulation of laser beams^14^, superluminescent diodes^15^, periodic variation of the vergence of a lens in the beam^16^, rotating diffusers^17^, and a low-cost one is the use of a vibrating mirror in the optical path^18^. To our knowledge, another typical speckle reduction technique, fiber shaking, has not been used so far in DP imaging. The method proposed in reference^18^ uses a vibrating mirror to scan the beam and descan it after reflection on the retina, such that the spot of light moves on the retina but not on the camera that is used for detection. Thus, using a long exposure time of the camera, the final DP image is the average over many images superimposed by different speckle patterns. A first disadvantage of this method is that moving parts increase the complexity of the system and are prone to malfunction because mechanical vibrations can, over time, misalign the optical components of the system. A second disadvantage is the long time needed to perform the measurements, which limits the applicability of the DP technique for real-time dynamic tests, which are needed, for example, for the clinical evaluation of tear film quality and the diagnosis of dry eye syndrome^19^.

Because speckle can be used to reconstruct the object from which the speckle pattern emerges^20–25^, one can wonder whether one can exploit speckle for reconstructing the PSF. However, the reconstruction will return the image of a point source in the retina, and will wash out the information that one aims to obtain from the DP image (the aberrations and scattering in the optics preceding the retina, which influence the light distribution on the retina and determine the actual optical quality of the eye).

Despite the fact that the speckle problem in DP images has received considerable attention, to the best of our knowledge no comparative study has yet been performed of the amount of speckle produced by different light sources. Here we aim to fill this gap by quantifying speckle in DP images acquired with a LD, a light emitting diode (LED), and a superluminescent diode (SLED), with and without a vibrating mirror in the optical path.

## Results

**Figure 1:**
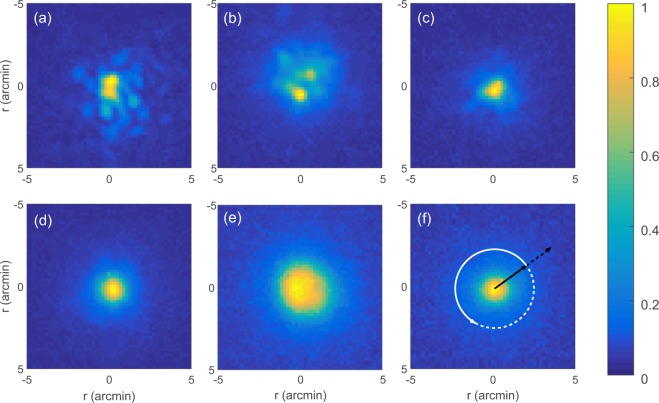
Point spread functions (PSFs). The color represents the intensity measured by the double pass camera (DPC in Fig. 6), normalized by the maximum value in each image. (**a**,**d**) LD, (**b**,**e**) LED, (**c**,**f**) SLED. Upper row: The vibration of the hot mirror (hMin Fig. 6) is turned off, and speckle patterns are observed on the images. Lower row: speckle is reduced when the vibration of the hot mirror is turned on.

Figure 1(a–c) display DP images obtained with the LD, the LED and the SLED, without further means of speckle reduction, while Fig. 1(d–f) display the DP images obtained using the same eye model and light sources, and turning on the vibration of the hot mirror. The DP images show that speckle formation is reduced by the vibration. Speckle is especially visible in the case of LD without the vibration, Fig. 1(a). As expected, speckle formation decreases when a spectrally broader source is used.

**Figure 2:**
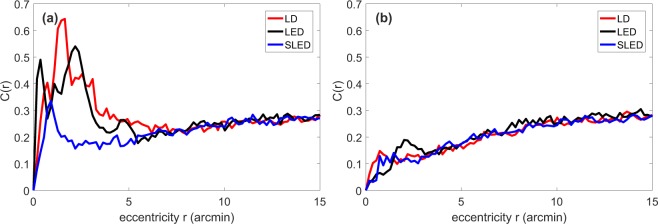
Radially evaluated speckle contrast (dimensionless) when the vibration of the hM mirror is turned off (**a**) and when it is turned on (**b**).

The radially resolved speckle contrast (see *Methods*), *C*(*r*), is shown in Fig. 2(a) and (b) without and with the vibrating mirror, respectively. Since there is only one pixel in the center of the PSF (and thus no variance), *C*(*r* = 0) = 0. While for small *r* the form of the curve depends on the shape of the speckle pattern overlying the PSF, for large *r* the value of the plateau is determined by the background noise. We attribute the peaks between *r* = 0 arcmin and *r* = 5 arcmin, especially visible in Fig. 2(a) to speckle.

In Fig. 2(b) we see that speckle is reduced to a similar degree for the three light sources when the vibrating mirror is used. In order to have a scalar quantifier, we compute the area under the curve from pixel 0 to 39 (that corresponds to *r*_0_ = 7 arcmin), which is when the lines of the different measurements take similar values, i.e., the start of the plateau. We refer to this index as *C*_1_. To verify that this approach gives consistent results, we also integrated *C*(*r*) over pixels 0–83 (i.e., up to *r* = 15 arcmin). We refer to this index as *C*_2_.

The values of *C*_1_ and *C*_2_ are listed in Table 1. Higher/lower values are due to more/less speckle present in the images. The case of the LD without mirror vibration shows the largest values because speckle is neither reduced by mirror vibration nor by broad band light. With the vibration on, we note that the three light sources cause a similar amount of speckle. Comparing the *C*_2_ values obtained with vibration, we see that the *C*_2_ value obtained with the LED is slightly higher than the *C*_2_ value obtained with the LD and SLED. This is likely due to the fact that the PSF of the LED is wider [see Fig. 1(e)]. Comparing the *C*_1_ or *C*_2_ values for SLED with no vibration and for LD with vibration, we can see that they are similar, which suggests that the SLED could be a good all-optical speckle-reduction solution, alternative to the vibrating mirror.

**Table 1:** Speckle quantification in the DP images; without/with mirror vibration. *C*(*r*) is integrated up to the limit of 7 arcmin, *C*_1_, and up to 15 arcmin, *C*_2_. After flattening the main PSF (see *Methods*) and subtracting the constant background, *C* is calculated in a square of 41 × 41 pixels (−3.7 to 3.7 arcmin), *C*_3_, and in a square of 181 × 181 pixels (−16.3 to 16.3 arcmin), *C*_4_.

	no vibration			vibration		
	LD	LED	SLED	LD	LED	SLED
*C*_1_	2.3	2.1	1.3	1.0	1.0	1.0
*C*_2_	4.3	4.1	3.3	3.0	3.2	3.0
*C*_3_	1.1	3.4	1.0	0.9	0.8	0.7
*C*_4_	1.1	1.8	0.7	0.7	0.6	0.5

The analysis of the DP images after removing the PSF and the background noise (see *Methods*) confirms this observation: both parameters, *C*_3_ and *C*_4_, indicate that the SLED with no vibration is almost as good as the LD with vibration. We note here high values for the LED without vibration, which we interpret as an artifact due to the fact that, in the DP image, the intensity does not have a well-defined center of mass [see Fig. 1(b)].

In order to investigate how the information extracted from the DP image (and used for diagnosis) is affected by the amount of speckle, we calculated the MTF for the three light sources with the vibrating mirror on or off by Fourier-transforming the double pass image. In the Fourier domain around zero spatial frequency, a peak appears, originating from a DC offset of the image due to retinal and ocular scattering of the model eye and other stray light, as well as camera noise. Therefore, the MTF values at other frequencies are scaled down. To evade this problem, we follow the method used in^2,26^, and replace the first two points of the MTF (up to 1.3 cyc/deg) by fitting the sum of two exponential functions, *f*(*ν*) = *C* ⋅ exp(−*Aν*) + *D* ⋅ exp(−*Bν*), with four parameters, to the remaining MTF data. An alternative for removing the DC peak is subtracting a constant background veil from the image before calculating its Fourier transform^27^.

To compare the MTFs, we first selected a benchmark measurement (as in^16^, we use the DP image recorded with the laser and the mirror vibrating). Then, we determine the relative difference of the MTF of a given configuration (M_*x*_), as a function of the spatial frequency *v*:

1 $${e}_{{\rm{M}}}(\nu )=\frac{{{\rm{M}}}_{x}(\nu )-{{\rm{M}}}_{b}(\nu )}{{{\rm{M}}}_{b}(\nu )},$$

where M_*b*_ is the benchmark MTF.

**Figure 3:**
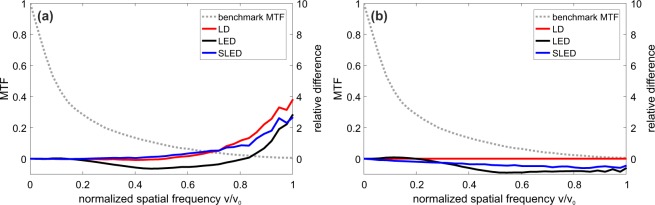
Benchmark modulation transfer function (MTF, in the left vertical axis, shown with dotted line), obtained by using the LD as light source and employing the vibrating mirror, vs. the spatial frequency, normalized to the cutoff frequency, *ν*_0_ = 44.75 cyc/deg. In the right vertical axis, the relative difference of MTF, Eq. (1), without (**a**) and with (**b**) mirror vibration compared to the benchmark MTF. In panel (b) the red line stays at zero because the LD with mirror vibration is used as the benchmark.

**Figure 4:**
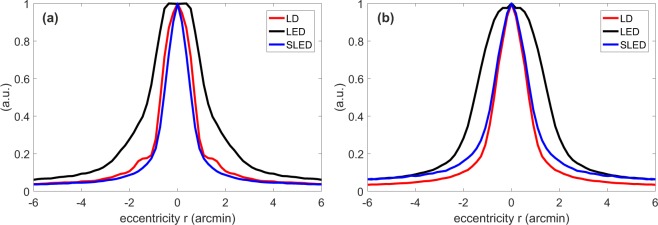
Radial profile of the PSF when the vibration of the mirror hM is turned off (**a**) and when it is turned on (**b**).

The results are presented in Fig. 3. Figure 3(a) shows the results for the measurements where the vibrating mirror was not used. The positive relative variations at higher frequencies are due to speckle present in the images. In Fig. 3(b), we see that when using the vibrating mirror, the relative variation is negative, because the vibrating mirror reduces the speckle. The red line, representing the LD, stays at zero because it is used as the benchmark. In both graphs, the MTF relative variation of the LED is negative (except in Fig. 3(a) at high frequencies). This is because the LED can not be considered a point source as narrow as the other sources, leading to a wider PSF, as shown in Fig. 4. The higher relative differences of the MTFs at higher normalized spatial frequencies in the case without the vibrating mirror (Fig. 3(a)) might be due to the roughness of the cardboard that acts as the retina in our eye model. For real eyes, the retinal cone spacing between 3 to 8 micrometers corresponds to spatial frequencies close to the cutoff frequency^28–31^.

## Discussion

We have done a comparative study of the amount of speckle in DP images recorded by using a laser diode (LD), a light emitting diode (LED) or a superluminescent LED (SLED) as light sources, with and without vibration of a mirror in the beam path. The setup allows for changing the light source without the need of realignment, thus permitting an objective comparison of DP images.

We have found that, when the mirror vibration is turned on, the three light sources yield very similar amounts of speckle. With the vibration turned off, the SLED gave a degree of speckle close to, but slightly higher than the degree of speckle obtained with the LD with vibrating mirror. The reduced speckle contrast of the SLED in comparison to the LD is due to its broader optical spectrum, which corresponds to a shorter coherence length of the light. Therefore, it can be an all-optical solution for speckle reduction in DP imaging, avoiding undesirable mechanical vibrations. The similar spectral widths of SLED and LED suggest comparable performance regarding speckle reduction. However, the light of the LED is difficult to collimate and experiences large losses when coupled to our setup by fiber, to the point of being too weak to obtain measurements of real eyes because of the required long exposure time. The SLED has a number of drawbacks, too: it is more expensive than the LD, and it does not reduce speckle to the extent of the LD with vibrating mirror.

The impact of longitudinal chromatic aberrations and diffraction on the DP images, corresponding to the light sources tested in this study, is negligible, as all them have peaks in the near infrared and limited spectral bandwidth (on the order of 50 nm for the LED and the SLED). In fact, differences of less than 1 pixel in the widths of the PSFs are expected from simulations.

As a low-cost, all-optical alternative it will be interesting to investigate if the light emitted by semiconductor lasers tailored to exhibit broadband, chaotic emission^32–34^ could be used to reduce speckle in DP imaging in a non-mechanical way. It will also be interesting, in future work, to investigate speckle reduction in DP images recorded from real eyes. Preliminary studies indicate that in a real eye, speckle is naturally reduced by the movements of the eye and micro-fluctuations of accommodation, making difficult to reliably estimate the reduction of speckle produced by either broadband illumination, or mechanical vibration.

## Methods

### Experimental setup

**Figure 5:**
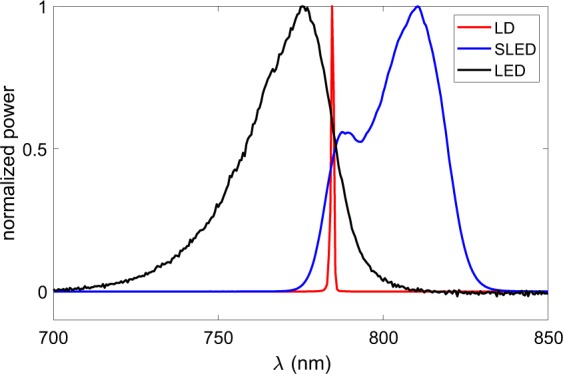
Normalized spectra of the light sources used.

**Figure 6:**
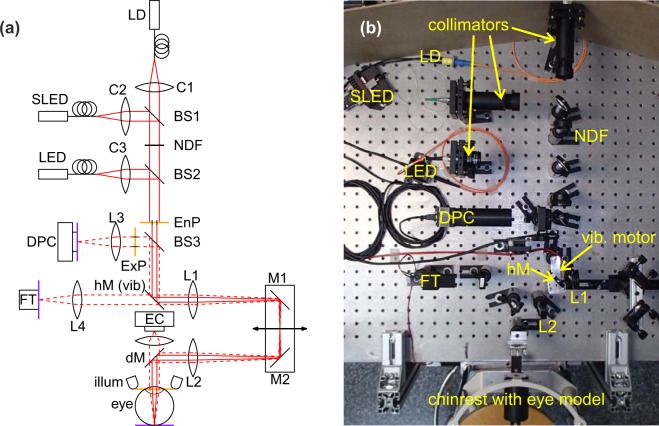
(**a**) Sketch of experimental setup. A laser diode (LD), a light-emitting diode (LED), and a superluminescent diode (SLED) are fiber-coupled into the optical setup and collimated by collimators C1–C3. NDF: neutral density filter. BS1 - BS3: 50% beam splitters, EnP/ExP: entrance/exit pupil, hM: hot mirror (with possibility to vibrate), dM: dichroic mirror, L1–L4: lenses (L1, L2: *f* = 150 mm, L3, L4: *f* = 100 mm) FT: fixation target, DPC: double pass camera, EC: eye camera, illum: eye illumination, eye: artificial eye. (**b**) Photo of the setup.

The experimental setup is depicted in Fig. 6. By using two 50% beam splitters, BS1 and BS2, the three light sources used (single-mode LD Monocrom 7850 MC with output power ~5 mW; Superlum SLED SLD 371 with ~6 mW, and Thorlabs LED M780F2 with ~1.15 mW) are fiber-coupled into the optical setup by three collimators (C1–C3) such that DP images can be compared without the need of realignment after changing the light source. As in clinical instruments, the optical power and wavelengths are chosen to meet the limitations of patient comfort, and therefore, the three light sources emit in the near-infrared. Their optical spectra (measured with an Instrument Systems Spectro 320 spectrometer), are depicted in Fig. 5. To couple light from the LED into the system, we used a 25 *μ*m diameter fiber as a trade-off between good collimation and intensity transmitted.

The collimated beam from the light source being used is guided to an eye model or to a real eye (eye), by a hot mirror (hM, with a motor attached which can be used to make it vibrate in order to vary the direction of the reflected beam by a small amount), a Badal system (consisting of two lenses (L1, L2, *f* = 150 mm each) and two mirrors, M1 and M2, mounted on a movable stage for correcting spherical refraction of the eye), and a dichroic mirror (dM, cutoff wavelength of 900 nm). The first pass of the light is indicated by red continuous lines. The eye model consists of a 50 mm lens and a black cardboard that mimics the retina (retinal plane and conjugated planes marked in purple). The eye camera EC (IDS UI-1240LE-M) was used to check the position of the eye, relative to the light beam. During positioning, the eye was illuminated with 940 nm LEDs (illum), which can pass the dichroic mirror (dM) to reach the eye camera (EC).

The entrance pupil (EnP) and exit pupil (ExP) of the system have diameters of 2 mm and 4 mm, respectively, and can be set by a diaphragm wheel. Both the EnP and ExP are placed in a plane conjugated to the pupil plane (marked in orange). We chose an entrance pupil diameter of 2 mm as a compromise between limiting the diffraction observed for smaller diameters and reducing the effect of aberrations of real eyes when using larger diameters^35^. After EnP, the intensity profile of the beam is approximately flat, which we checked with a camera (IDS UI-1240SE-M) at the pupil plane of the eye.

According to^36^, to reach diffraction limit with a 2 mm entrance pupil, visual correction has not only to be performed for defocus, but also for astigmatism. Since we are mainly interested determining the amount of speckle in our DP images, and use an eye model with negligible spherical and cylindrical aberrations, we do not consider the correction of astigmatism here.

After its second pass in reverse direction through the eye and through the Badal system, the light beam is coupled out by a beam splitter, BS3 (50%), and imaged by a lens of *f* ′ = 100 mm focal length onto a CMOS camera (DPC, IDS UI-1240SE-M, 5.3 *μ*m × 5.3 *μ*m pixel size). All measurements were performed with the same exposure time of 500 ms which was chosen in order to fill the dynamic range of the camera, even with the least powerful of the sources, the LED. In the measurements performed with the eye model, the power of the LD and the SLED were adjusted by the driving current and a neutral density filter (NDF, absorbance = optical density 2), in order to provide similar power as the LED at the corneal plane, i.e. ~0.02 *μ*W. The main source of losses is the bad coupling of the LED to the fiber.

The setup also includes a fixation target, FT, that is aimed at helping future patients to fixate their view at infinity.

### Quantification of the speckle contrast

To quantify the amount of speckle, the speckle contrast, $C={\sigma }_{I}/\langle I\rangle$ (where $\langle I\rangle$ is the mean intensity of the pattern and *σ*_*I*_ is its standard deviation), is the standard measure used in full-field imaging, laser projection, microscopy, etc.^12,37–39^. This measure is useful when the speckle pattern is spatially homogeneous, but it is not useful for DP images, where the PSF, superimposed by speckle, is localized in the center of the image (see Fig. 1).

Thus, assuming PSF azimuthal symmetry, we calculate the radial variation of the speckle contrast^16^, as shown schematically in Fig. 1(f): $C(r)={\sigma }_{I}(r)/\langle I(r)\rangle$, where *σ*_*I*_(*r*) and $\langle I(r)\rangle$ are computed along a circle of radius *r*, centered at the center of mass of the intensity.

In addition, for each image we performed the following analysis aimed at removing the PSF and the constant part of the background noise before computing the speckle contrast: for all pixels at distance *r* from the center, we determined the minimum intensity value, *I*_*min*_(*r*), and obtained a new image by subtracting *I*(*r*) − *I*_*min*_(*r*) (we use the minimum instead of the average to avoid negative values). Then, we calculated the speckle contrast in the central square of the image where speckle is concentrated (41 × 41 pixels), *C*_3_, and in a larger region (181 × 181 pixels), *C*_4_, where the speckle contrast measure is more affected by non-constant background noise.

## Data Availability

The data is available on request, please contact the corresponding author.
